# Clinical Efficacy and Dosing of Vibrotactile Coordinated Reset Stimulation in Motor and Non-motor Symptoms of Parkinson's Disease: A Study Protocol

**DOI:** 10.3389/fneur.2021.758481

**Published:** 2021-11-18

**Authors:** Kristina J. Pfeifer, Alex J. Cook, Jessica K. Yankulova, Bruce J. P. Mortimer, Elizabeth Erickson-DiRenzo, Rohit Dhall, Leila Montaser-Kouhsari, Peter A. Tass

**Affiliations:** ^1^Department of Neurosurgery, Stanford University School of Medicine, Stanford, CA, United States; ^2^Engineering Acoustics, Inc., Casselberry, FL, United States; ^3^Department of Otolarygology Head and Neck Surgery/Laryngology Division, Stanford University School of Medicine, Stanford, CA, United States; ^4^Department of Neurology, Center for Neurodegenerative Disorders, University of Arkansas for Medical Sciences, Little Rock, AR, United States; ^5^Department of Neurology and Neurological Sciences, Stanford University School of Medicine, Stanford, CA, United States

**Keywords:** coordinated reset, vibrotactile stimulation, Parkinson's disease, study protocol, sensorimotor, non-motor symptoms, non-invasive stimulation

## Abstract

Enhanced neuronal synchronization of the subthalamic nucleus (STN) is commonly found in PD patients and corresponds to decreased motor ability. Coordinated reset (CR) was developed to decouple synchronized states causing long lasting desynchronization of neural networks. Vibrotactile CR stimulation (vCR) was developed as non-invasive therapeutic that delivers gentle vibrations to the fingertips. A previous study has shown that vCR can desynchronize abnormal brain rhythms within the sensorimotor cortex of PD patients, corresponding to sustained motor relief after 3 months of daily treatment. To further develop vCR, we created a protocol that has two phases. Study 1, a double blinded randomized sham-controlled study, is designed to address motor and non-motor symptoms, sensorimotor integration, and potential calibration methods. Study 2 examines dosing effects of vCR using a remote study design. In Study 1, we will perform a 7-month double-blind sham-controlled study including 30 PD patients randomly placed into an active vCR or inactive (sham) vCR condition. Patients will receive stimulation for 4 h a day in 2-h blocks for 6 months followed by a 1-month pause in stimulation to assess long lasting effects. Our primary outcome measure is the Movement Disorders Society-Unified Parkinson's Disease Rating Scale (MDS-UPDRS) part III off medication after 6 months of treatment. Secondary measures include a freezing of gait (FOG) questionnaire, objective motor evaluations, sensorimotor electroencephalography (EEG) results, a vibratory temporal discrimination task (VTDT), non-motor symptom evaluations/tests such as sleep, smell, speech, quality of life measurements and Levodopa Equivalent Daily Dose (LEDD). Patients will be evaluated at baseline, 3, 6, and 7 months. In the second, unblinded study phase (Study 2), all patients will be given the option to receive active vCR stimulation at a reduced dose for an additional 6 months remotely. The remote MDS-UPDRS part III off medication will be our primary outcome measure. Secondary measures include sleep, quality of life, objective motor evaluations, FOG and LEDD. Patients will be evaluated in the same time periods as the first study. Results from this study will provide clinical efficacy of vCR and help validate our investigational vibrotactile device for the purpose of obtaining FDA clearance.

**Clinical Trial Registration:** ClinicalTrials.gov, identifier: NCT04877015.

## Introduction

More than 6.1 million people suffer from Parkinson's Disease (PD) worldwide (1), making it the most widespread neurodegenerative disorder second to Alzheimer's Disease (2). Dopamine precursors, such as levodopa, are used in medication to treat PD and are considered the gold standard in improving motor function (3). However, given that PD is a neurodegenerative disease resulting in patients' symptoms worsening over time, dopaminergic therapy can only go so far before patients increase their medication or no longer feel the same therapeutic benefit (3, 4). Furthermore, medications that increase dopaminergic transmission can have unwanted side effects, such as vomiting, hypotension, delusions, and dyskinesia (5). In addition, dopaminergic induced psychosis is often reported in PD patients, especially those in the advanced stages of the disease who experience cognitive impairment (6). In the later stages of PD, patients may undergo Deep Brain Stimulation (DBS), which has demonstrated to be more effective than medication alone (7). However, the invasiveness of the procedure and related potential surgical side effects (e.g., hemorrhage) (8) as well as side effects of the chronic stimulation, considered as DBS-induced movement disorders (9), make it less appealing to patients. For instance, different types of dyskinesias, blepharospasm, and apraxia of eyelid opening were observed with DBS delivered to the subthalamic nucleus (STN), whereas hypokinesia and freezing of gait were described with DBS of the globus pallidus internus (GPi) (9). Furthermore, DBS delivered to standard targets for PD, such as the STN or GPi, is less effective in treating gait, balance (10), and dysarthrophonia (11). Even in conjunction with medication, traditional high frequency DBS only provides temporary motor improvement, with symptoms returning almost immediately after cessation of stimulation (12). The development of non-invasive therapies that improve PD symptoms and potentially change pathological PD brain states in a way which slows, or reverses disease progression is much needed and essential in overcoming the limitations of the two most common types of PD treatments (13).

Abnormal neuronal synchrony of beta band is often found within the STN of PD patients (14), with decreases in this band correlating with improved motor capability (15). Based on this finding, we developed vibrotactile Coordinated Reset (vCR) which is a non-invasive treatment that delivers weak, non-painful random vibrations to the fingertips of patients (16). This type of therapy is based on extensive computational research done on the desynchronizing effects produced by CR (17–19). CR stimulation aims at disrupting neuronal synchronization by delivering phase resetting stimuli, typically periodically in time, separated by equidistant time differences given by T_s_/N_s_, where T_s_ is the duration of a stimulation cycle, and N_s_ is the number of active stimulation sites (20). Computationally, it was shown that CR-induced desynchronization may cause a reduction of the rate of neuronal coincidences, and in turn, a decrease of the strength of plastic synapses, ultimately shifting neural networks from stable, synchronized, strongly synaptically-connected states to stable desynchronized states with weak connectivity (18–21). For this, CR uses spike-timing-dependent plasticity (STDP), a fundamental learning mechanism that adapts the strength of synapses based on the relative timing of their pre- and postsynaptic spikes or bursts (22). Furthermore, in this study, we consider a vCR pattern with moderate stimulus time jitter. This is motivated by a previous computational study introducing spatial and temporal jitter (21). To this end, random reset (RR) stimulation was administered to a network of leaky integrate-and-fire (LIF) neurons with STDP and electrical model stimuli (21). It was shown that RR stimulation, characterized by adding spatial and temporal noise to the mechanism of CR stimulation, may lead to more robust long-term desynchronizing effects, that are less dependent on the detuning of the mean inter-stimulus interval in comparison with the dominant frequency of the abnormally synchronized neuronal rhythm (21).

In a previous study, monkeys injected with 1-methyl-4-phenyl-1,2,3,6-tetrahydropyridine (MPTP) received brief, high frequency electrical pulse trains that were administered to the STN (CR-DBS) for 2 h on five consecutive days (23). Acute and sustained motor improvement lasting for several weeks was observed (23). This study was then performed with PD patients who received CR-DBS administered to the STN for 4 h a day for three consecutive days. Reduced beta band synchronization occurred, which correlated with a significant improvement of motor ability (24).

Similar findings have also been documented using vibration as a CR stimulus (17, 25). The first-in-human vCR study was conducted with five idiopathic PD patients, who received vCR stimulation for 4 h a day over three consecutive days (25). Patients exhibited improvements in gait and bradykinesia both during stimulation and after a 1-month pause in stimulation. Vibration by itself is known to increase motor responsiveness (26) and activation of the sensorimotor cortex (27) which corresponds to a decrease in cortical alpha and beta power (27, 28). In our most recent work, we further optimized vCR stimulation patterns and parameters which led to significant improvements in motor ability in two PD studies (17). Study 1 consisted of a 3-month vCR intervention, in which patients received stimulation for 4 h a day. At baseline and after 3 months of vCR therapy, we examined motor changes in the Movement Disorders Society Unified Parkinson's Disease Rating Scale part III (MDS-UPDRS) (29) and recorded electroencephalography (EEG) beta band activity while patents were at rest (17). After 3 months of daily vCR treatment, PD patients off medication and at rest exhibited a cortical decrease in high beta band (21–30 Hz) power in the sensorimotor cortex compared to baseline (17). Additionally, patients showed significant improvements on the MDS-UPDRS III while off medication both acutely (after 4 h of stimulation at day one) and cumulatively (after 3 months of daily vCR therapy) (17). Furthermore, by the 3rd month, patients were able to decrease their Parkinson's medication by an overall 7.82% (17). Study 2 examined the cumulative motor effects in a three-patient case study in which patients received daily vCR stimulation for 6+ months (17). MDS-UPDRS assessments were performed every 3 months while off medication. All patients showed a significant improvement in their motor ability (17). Additionally, of the three patients, one maintained their current medication regimen pre-study, while the other two reduced their medication (10.86 and 66%, respectively) by the end of the study (17). In one patient, we planned a 1-month pause in stimulation after 6 months of therapy. Results showed no considerable differences in motor ability. Additionally, we reduced this patient's daily vCR sessions (4 h) to 2 h three times a week after the 7-month follow-up. The patient continued to show significant motor improvements for the remainder of the study (3 additional months) (17).

Taken together, we believe that vCR has the potential to drastically improve motor abilities during and post treatment, decrease patient medication intake and potentially slow disease progression in a way that is non-invasive and presents little to no side effects. The current study protocol: *Vibrotactile coordinated reset: a non-invasive treatment for Parkinson's disease*, aims to understand the therapeutic benefits of vCR in a larger sample size, test against a dedicated sham pattern, study vCR effects on not only motor outcomes, but voice, speech, sleep and other non-motor symptoms, study vCRs long-lasting effects, and finally a vCR dosing regimen all within a 14-month clinical study protocol. Together, results from this study will demonstrate clinical efficacy of our vCR stimulation pattern and vibrotactile device for the purpose of acquiring Food and Drug administration (FDA) clearance.

## Methods and Analysis

### Vibrotactile Coordinated Reset Therapy

**Figure 1:**
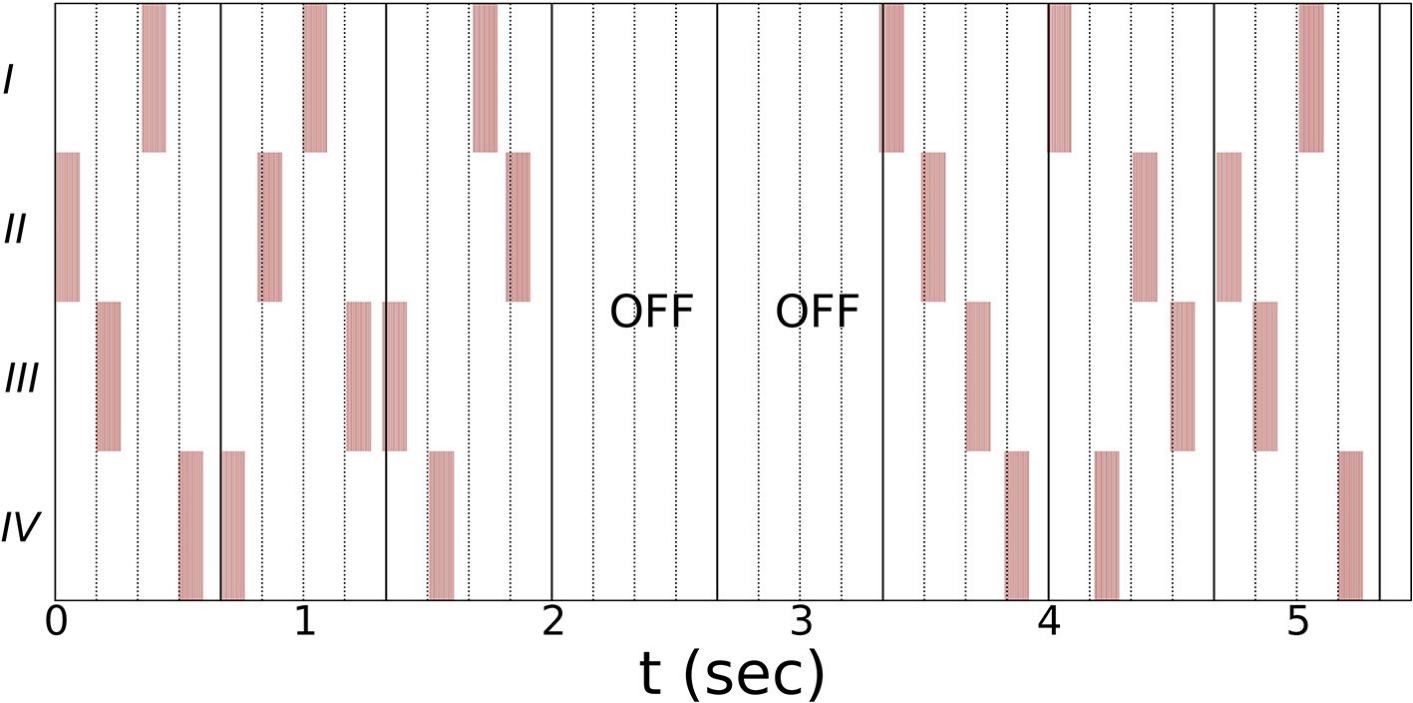
Schematic illustration of the vCR stimulation using a three cycles ON: two cycles OFF pattern (17). Single vibratory bursts (highlighted by red bars) are delivered at periodic times subjected to a jitter that is uniformly distributed within the range of ±23.5% the inter-stimulus intervals. A vCR sequence comprises four subsequent vibratory bursts, delivered (on average) within one vCR cycle. Within one vCR sequence each fingertip (two through five) is activated exactly once. The CR frequency *f*_*CR*_, i.e., the rate at which the CR sequences are delivered, is 1.5 Hz. Hence, the length of a CR cycle is 667 ms. Bilateral noisy vCR is administered in a mirrored manner to both hands, coincidently activating right and left fingers (two through five). Schematic shows the vCR pattern coincidently delivered to left and right hand. Vibration frequency is 250 Hz and duration of vibration bursts is 100 ms. The duration of a single vCR session is 2 h.

Noisy vCR stimulation (see Figure 1 for a schematic representation) was introduced by Pfeifer et al. (17). Vibratory stimuli are delivered at periodic times with a jitter that is uniformly distributed within the range of ±23.5% the inter-stimulus intervals. A vCR cycle comprises a sequence of four vibratory bursts delivered to each fingertip. The vCR sequence is randomly varied from one cycle to another. Three cycles with vCR stimulation ON are followed by two cycles with vCR turned OFF (3:2 ON–OFF CR). We apply bilateral noisy vCR in a mirrored manner, such that the right and left fingers two to five get coincidently activated, respectively. Vibration frequency is 250 Hz and duration of vibration bursts amounts to 100 ms. As in Pfeifer et al. (17) the CR frequency (*f*_*CR*_), which is the CR sequence delivery rate, is 1.5 Hz. Accordingly, the length of a CR cycle is 667 ms. The duration of a single vCR session amounts to 2 h. To avoid patient unblinding, the parameters for the sham stimulation will be presented in our clinical results paper upon completion of this study.

### Vibrotactile Device Description

**Figure 2:**
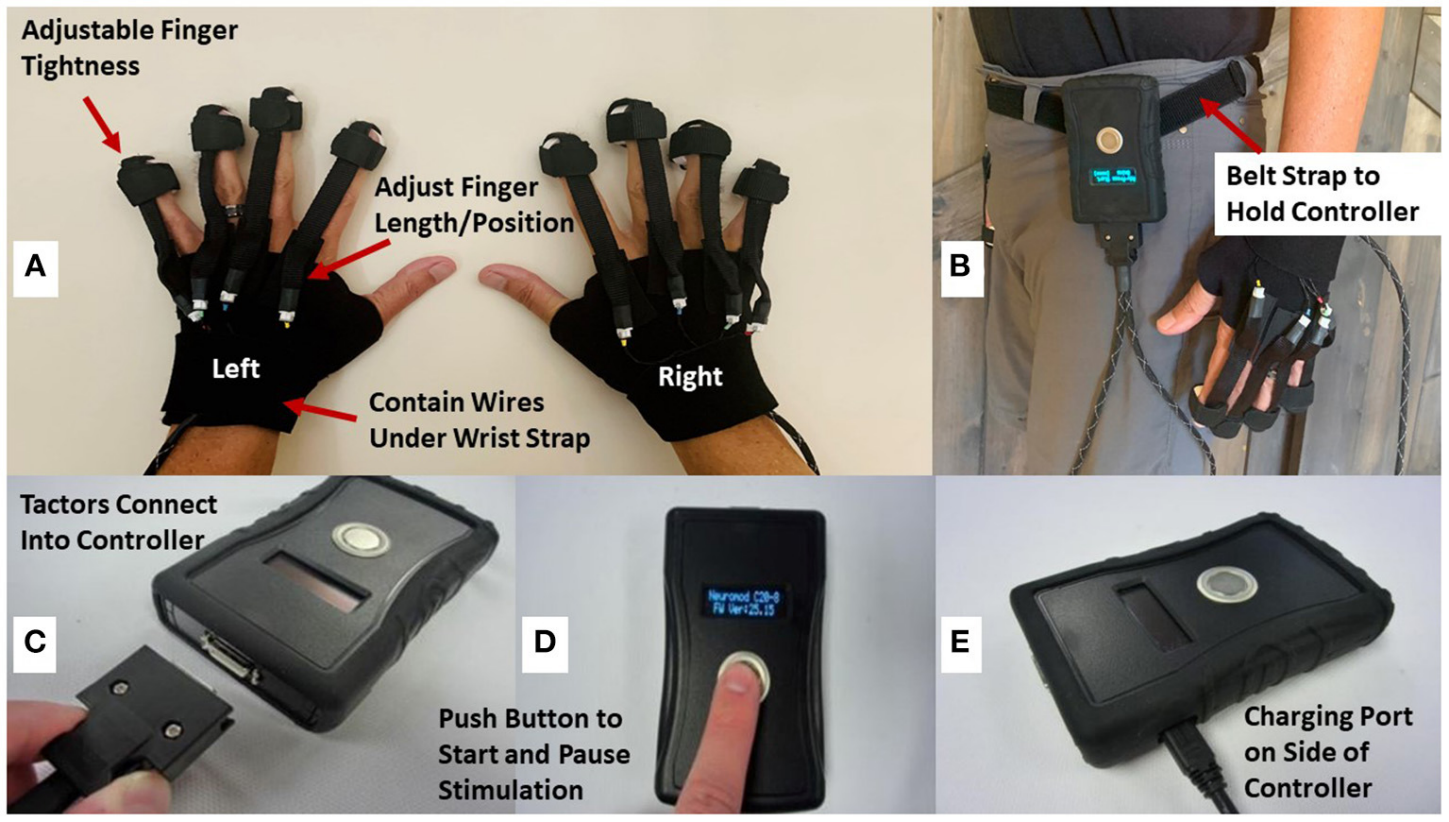
Displays the vibrotactile wearable stimulator. **(A)** Displays the tactors and gloves that can be adjusted *via* Velcro straps for the finger pad or length of finger. **(B)** Depicts the controller being worn and fastened by a belt. **(C)** Displays the part of the controller in which the tactor ends plug into. **(D)** Displays the LCD screen which shows battery life and time remaining of stimulation. A push button turns on, pauses, and turns off the controller. **(E)** Displays the charging port of the controller which is charged with a micro-USB.

The vibrotactile device is investigational and has not yet been cleared by the FDA for clinical use. The vibrotactile device is a mobile, battery-operated controller with wire-connected vibrotactile stimulators (tactors) fastened onto the fingertips of a custom glove (see Figure 2). The glove is fitted by trained clinical research coordinators during the baseline visit. Both sham and active vCR stimulation patterns were developed by the study's principal investigator and were tested together with Engineering Acoustic Incorporated (EAI). Stimulus patterns are loaded into the controller *via* secure digital (SD) cards. The controller is small enough to fit into a pocket or can be fastened to a belt (Figure 2B). The tactors (Figures 2A,B) are connected to a glove and are individually fastened to the fingertips *via* elastic Velcro bands. The tactors are connected to the controller (Figure 2C) and an Organic Light-Emitting Diode (OLED) screen displays information about battery status and the time left in the therapy session. The controller has a push button that can start, pause or turn off the device (Figure 2D). The controller logs patient therapy sessions and stores this information on the SD card. The research personnel uses the log information to verify that each patient is stimulating according to the stimulation protocol procedures. The controller has a charging port on its side (Figure 2E) and is charged with a micro-USB.

### Patient Population

Patients will be screened and selected from the population of patients presenting with idiopathic PD who are routinely seen in the Stanford Neuroscience Clinic, referred from non-Stanford clinics or have found this study through clinicaltrials.gov. The principal investigator, designated movement disorders neurologist and research coordinators may introduce the study to potential candidates in-person at Stanford's Neuroscience Clinic. Additionally, clinical research coordinators may also contact potential candidates by phone or email after the referral. Patients allowed into the study will only be from the San Francisco Bay area.

### Inclusion and Exclusion Criteria

Patients included in this trial will be between 45 and 85 years of age and have a diagnosis of idiopathic Parkinson's disease with Hoehn and Yahr Stages II–IV while on medication. Patients will need MDS-UPDRS III motor improvement ≥30% while on medication compared to while off medication and be on stabilized medication. Patients cannot have dopamine dysregulation syndrome or presence of other neurological diseases such as major depression, dementia, attention deficit disorder, psychosis, or essential tremor. Patients cannot have a history of epilepsy, traumatic brain injury or brain surgery. Patients cannot have severe sensory abnormalities of the fingertips such as vibratory urticaria. Patients must clearly communicate with staff and speak English. Patients are excluded if they are currently on psychoactive or narcoleptic medications or are on medications that affect brain function or alter EEG recorded activity (i.e., anticonvulsants, ADHD, depression, or anxiety medication). Participation in this study requires that all patients do not participate in another drug, device, biologic, or intervention trial concurrently or within the preceding 30 days. Patients who are pregnant, breastfeeding, or trying to get pregnant during the duration of the study are excluded. Lastly, San Francisco Bay area residents may only be included in this study.

### Pre-assessment Measures for Inclusion of Clinical Trial

Prior to on-site assessment measures, patients will receive a General Health Survey which includes questions regarding health history, inclusion/exclusion criteria and questions relating to dopamine dysregulation syndrome. If the patient is considered a good candidate, a trained staff neurologist, specialized in movement disorders, will verify idiopathic PD in potential patients at an on-site pre-study visit, four-eight weeks before the initial trial. Depending on the type(s) of Parkinson's medication the patient takes, he or she will be asked to withdraw from medication (12–48 h) prior to the in-person assessments. On-site, patients will be asked about their health history and a series of neurological and physical examinations will be performed by the study's movement disorders neurologist to rule out patients with physical or neurological problems unrelated to PD that may impact the study results. Verification of motor responsiveness to dopaminergic medication will be assessed using part III (motor evaluation) of the MDS-UPDRS. Patients will arrive off medication and perform the MDS-UPDRS III. Patients will then be prescribed Parcopa, a carbidopa-levodopa orally disintegrating, fast acting tablet that takes ~1 h to take effect after which the MDS-UPDRS III will be performed again. Patients will then additionally perform the Mini Mental State Examination (MMSE) (30) and Scales for Outcomes in Parkinson's disease-Cognition (SCOPA-COG) (31) to rule out PD dementia. To exclude patients with severe vibratory sensory abnormalities, such as vibratory urticaria, patients will receive vibrations with high (0.35 mm) and low (0.03 mm) peak vibration amplitudes to each individual fingertip and be asked to verify which vibration type they received. Each patient's medication is then tracked for 1 month by the movement disorders neurologist to confirm that the patient's medication is stable.

### Study Personnel and Their Roles

This study will include five movement disorders neurologists who are blinded during the first study. The first movement disorders specialist will serve as the *treating movement disorders neurologist* and perform physical and neurological examinations, evaluate and provide medical advice regarding patients' medication intake and serve as point of contact in the event of a serious or adverse event either unrelated or related to vCR for both the first and second study. The second movement disorders neurologist will serve as the studies' *main MDS-UPDRS movement disorders evaluator* who will perform assessments on patients throughout the first and second study. Three other movement disorders specialists will serve as the study's *video MDS-UPDRS III raters* for the first (main) study. The clinical research coordinators will oversee consent, objective motor measurements, video recordings, patient contact, vibrotactile glove administration, EEG recordings, voice recordings, and all self-report tests and questionnaires in both studies. Speech analysis will be performed by a voice disorders specialist and her trained team. An un-blinded statistical analyst, who is not associated with the study team and does not report to any member of the study team, will perform all statistical tests on outcome measures. All movement disorders specialists, speech analysis, and clinical research coordinators, will be blinded until completion of the first study.

### Study 1 Design: Main Phase—Double Blind Sham-Controlled

We will perform a 7-month, double-blind, sham-controlled study including 30 PD patients randomly placed into either active vCR (*n* = 15) or a sham (*n* = 15) condition. All personnel and PD patients will be completely blinded to which stimulation pattern patients obtain. Parkinson's patients will receive vCR or sham stimulation for a total of 4 h a day (2 times 2 h a day with a break in between the 2-h sessions) at home for 6 months. To measure long-term effects, patients will pause stimulation for 1 month after the 6-month follow-up appointment.

#### Assessments: Study 1

The following outcome measures and their descriptions will be administered to patients. The *MDS-UPDRS parts IA, IB, II, III*, and *IV* (29). The MDS-UPDRS parts IA and IB concerns non-motor experiences of daily living, in which IA is assessed by the study's main MDS-UPDRS movement disorders evaluator and IB is self-reported. Part II is motor experiences of daily living and is self-reported. Part III is the in-person motor evaluation assessed by the study's main MDS-UPDRS movement disorders evaluator. In addition, part III will be video recorded and sent to the three blinded video MDS-UPDRS III raters. Patient recordings will be evaluated after each patient completes his or her 7-month visit. Video raters will additionally be blinded to the date of administration. Part IV incorporates patient details on motor complications with the study's main MDS-UPDRS movement disorders evaluators observations and judgements. The *PD Quality of Life Questionnaire-39* (*PDQ-39*) (32) is a self-report questionnaire that examines health related difficulties specific to PD in eight quality of life categories within the last month. The *Parkinson's disease sleep scale* (*PDSS-2*) (33) examines PD related sleep issues during the past week. The *University of Pennsylvania Smell Identification Test* (*UPSIT*) (34) is a smell test comprised of 40 odors, in which patients try to correctly identify the odorant presented. Patients will also take a tolerability and usability questionnaire regarding the vCR device. We will perform a *vibratory temporal discrimination task* (*VTDT*) that consists of two vibratory bursts, with one burst delivered to the index finger and one burst to the middle finger. Each burst will start randomly on either the index or middle finger. This procedure will be performed on the right and left hand separately. The patient is instructed to judge if he/she felt a delay between the two vibratory bursts. This task was designed as a possible calibration method for future vCR studies by serving as a sensitivity measure for vibratory temporal changes, in which reduced perceived vibratory time differences correspond to increased vibratory temporal discrimination. Patients will also undergo clinically established *speech and voice assessments*. Speech samples will be collected at a sampling rate of 44,100 Hz on a laptop using the Praat Speech Analysis program (Version 5.4, University of Amsterdam). To collect samples, a head-worn, unidirectional microphone will be placed over the participant's ears and the microphone will be adjusted so that it is 6 cm from the participant's mouth. Specific samples will include sustained vowel phonations, sentence and paragraph length reading passages, and spontaneous speech. From these samples, speech and voice assessments will be conducted including measures of articulatory precision, speech intelligibility, speech rate, auditory-perceptual ratings of voice, and acoustic measures of vocal fundamental frequency, vocal intensity, and fundamental frequency and intensity variability. Additionally, the sentence intelligibility portion of the Assessment of Intelligibility in Dysarthric Speech (AIDS) (35) and patient self-assessment scales of Communicative Participation Item Bank (CPIB) (36) and the voice handicap index (VHI) (37) will be collected. For objective measures, patients will perform the *Kinesia ONE* motor evaluation, which uses a wearable accelerometer to assess motor activities similar to the MDS-UPDRS III and record their Parkinson's medication intake. The *Ambulatory Parkinson's Disease Monitoring* (*APDM*)'s Mobility Lab system will be used to measure objective gait disturbances. Lastly, the patient will also complete three different types of tasks during which *EEG* will be recorded. The first is a *sensorimotor EEG task*, in which patients receive a single vibratory stimulus to a random finger (excluding the thumb) on their non-dominant hand and are instructed with their dominant hand to push the response pad as fast as possible when they feel the vibratory burst. Each finger receives an equal number of vibratory pulses (50 per finger, equaling 200 trials) in a randomized order. Cortically, we expect to look at motor evoked potentials in response to cued vibration and their amplitude and latency changes throughout the course of treatment. Reaction time will also be documented. Additionally, we will record *vibration-only evoked potentials* in which we look at how two different types of vibratory pulses (high- and low- amplitude) affect different motor and sensory areas of the brain. The last task will be a recording done while the patient is at rest (*spontaneous EEG*). Patients will receive either active vCR or sham depending on the condition to which they were randomly assigned. For this task, we want to replicate our previous finding of decreased high beta band power (21–30 Hz) (17) for patients who received real vCR and quantify differences in activated brain areas in response to sham or active vCR. The MDS-UPDRS III off medication will be used as our primary outcome measure. All other measures are considered secondary.

#### Study 1: Visit Procedures

After inclusion into the study, the patients will be pseudo-randomly placed into a vibrotactile sham or real vibrotactile condition by staff personnel, not affiliated with the study. Patients will take the MDS-UPDRS parts IB, II, PDQ-39, PDSS, and the UPSIT online and at home on medication 1–2 weeks before every study visit. Patients will be asked to complete a usability and tolerability questionnaire about the vCR device after receiving therapy for 1 week following the start of the trial. Patients will then retake this questionnaire 1–2 weeks before all subsequent study visits.

Study visits will occur at baseline, 3, 6 months, and after a 1-month pause in stimulation at 7 months. Patients will arrive off medication and perform the following assessments in order. The participant's health history, physiological and neurological state will be examined upon arrival. Then, patients will perform the MDS-UPDRS III and will be video recorded. Electroencephalography recordings, the VTDT and speech assessments will be administered. Patients will then perform objective measurements including Kinesia ONE and the APDM. Parcopa is then given, and patients receive a 1-h break. The following assessments will be done on medication following the 1-h break. The MDS-UPDRS parts IA, III, IV, Kinesia ONE, and the APDM.

#### Study 1: At Home Therapy Procedures

**Figure 3:**
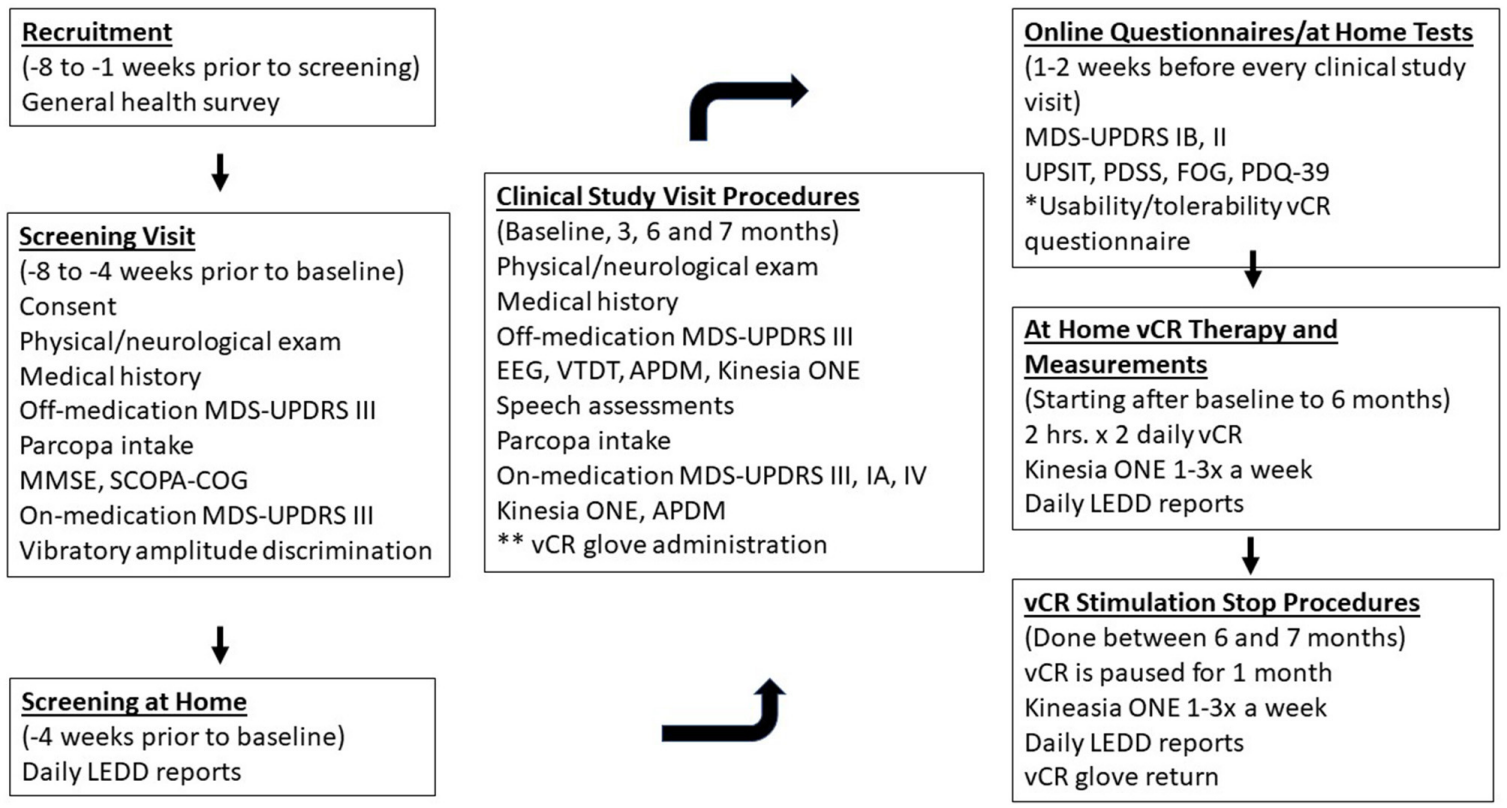
Depicts study 1 procedures for the double-blind, sham-controlled vCR study. Patients will receive a General Health Survey which includes our inclusion/exclusion questionnaire and our dopamine dysregulation syndrome questionnaire developed for the purpose of our study *via* email 8–1 weeks prior to the screening visit. During the screening visit (8–4 weeks prior to baseline) patients will be consented and will arrive at Stanford off medication, to complete their physical/neurological examination and report their PD medical history. Patients will perform the MDS-UPDRS III off medication with the study's main movement disorders evaluator and then receive Parcopa (dopamine medication). Patients will then repeat the MDS-UPDRS III after 1 hour of taking medication. Patients will complete a vibratory intensity discrimination task to confirm that they do not have severe vibratory sensory abnormalities. The study's treating movement disorders neurologist will track patients for 4 weeks prior to their first clinical study visit (baseline) to confirm that their medication is stable. One to two weeks prior to every clinical study visit, patients will receive the following questionnaires/tests: MDS-UPDRS IB, II, UPSIT, PDSS, FOG, PDQ-39, and the usability/tolerability questionnaire of the vCR device. During the clinical study visits (baseline, 3, 6, and 7 months), patients will arrive to Stanford off medication and undergo the physical/ neurological examination, PD medical history, MDS-UPDRS III, EEG, VTDT, APDM, Kinesia ONE, and speech assessments. Patients are administered Parcopa and after 1 h perform the MDS-UPDRS III, IA, IV, Kinesia ONE, and APDM. After the 6-month visit, patients undergo a 1-month pause in stimulation to measure long term effects of vCR at the 7-month follow-up. During the entire length of the trial, patients will report their daily LEDD amount and starting from baseline will perform the Kinesia ONE motor evaluation at home one to three times a week for motor monitoring. *The Usability/Tolerability questionnaire is performed 1 week after the start of therapy and then 1-2 weeks before every study visit. **vCR glove administration occurs at baseline only.

During the first study visit, patients will be taught how to use, wear and adjust the vibrotactile device. Patients will then be sent home and asked to stimulate for a total of 4 h a day with a break in between each 2-h session (minimum 1-h break) for 6 months. At the end of the 6 months, patients will be asked to stop stimulation for 1 month. During the 7-month study, patients are asked to continue their prescribed medication as needed. If the patient would like to decrease his or her medication due to positive motor results throughout the study, the patient can reduce medication according to the advice of our study's treating movement disorders neurologist and the patient's personal movement disorders neurologist. While at home, patients will have their own Kinesia ONE system to log medication information and perform motor tasks while on medication 1–3 times every week to monitor movement ability. After the start of the study, patients will have a 1-month checkup with the study's treating movement disorders neurologist over the phone to check in on how the participant is doing. If the participant experiences any worsening of motor ability/or side effects that he/she believes is due to the vibrotactile device after the 1-month checkup, the patient can schedule a phone call with the study's treating movement disorders neurologist to discuss what the next steps will be. In the event the participant experiences an adverse event or a serious adverse event either related or unrelated to glove, he/she is required to schedule a call with the study's treating movement disorders neurologist for evaluation within 3 days of the event. For a detailed schematic of study 1 events see Figure 3.

#### Study 1: Statistical Analysis and Anticipated Results

A sample size of 30 was selected as a starting number of patients for the clinical trial. A priori analysis indicated that for a sample size of 30, we would need a large effect size (*f* = 0.42) in order to reach statistical significance (*p* ≤ 0.05). In our previous study of six patients receiving vCR treatment for 3 months, using a paired samples *t*-test to compare MDS-UPDRS III scores pre- and post-treatment, the effect size was large (*d* = 1.011) (17). Based on this, our large *f* effect size of 0.42 may be reasonable. Nevertheless, an interim analysis will be performed by an un-blinded, experienced statistical research personnel, who is unaffiliated with the study team and does not report to any study team member, the principal investigator, nor any neurologist involved in the studies. This interim analysis will be performed when 16 patients have completed the 3-month mark, with eight belonging to the sham group and eight belonging to the vCR group. Results from the interim mixed factorial analysis of variance (ANOVA), will allow us to recalculate our desired sample size.

A 2 (sham vs. real vCR) by 4 (baseline, 3, 6, and 7 months) mixed factorial ANOVA will be done separately on the following measures: MDS-UPDRS I, II, III, IV, Levodopa equivalent daily dose (LEDD), EEG sensorimotor related evoked components, vCR resting EEG beta power response, vibratory temporal discrimination, speech assessments, UPSIT, PDSS, PDQ-39, FOG, and gait measurements obtained from the APDM Mobility Lab.

Statistically, for patients who received real vCR, we hope to see significant improvement in all measures by 6 months and expect to see no significant worsening when comparing assessments done after the pre-planned 1-month pause in stimulation at 7 months. In addition, we expect to see no significant improvements in the sham group patients.

### Study 2 Design: Second Phase Remote Dosing Regime

At the 7-month appointment, the patient will be unblinded and given the option to continue or start real vCR stimulation for 6 additional months followed by a 1-month pause in stimulation. During the 7 additional months, patients will be remotely monitored and instructed to stimulate for six additional months as needed with parameters set to 2 h a day (maximum daily dose) to 2 h a day three times a week (minimum weekly dose). In addition, long-term effects will be measured after a 1-month pause in stimulation following the 6 additional treatment months.

#### Remote Study Procedures

**Figure 4:**
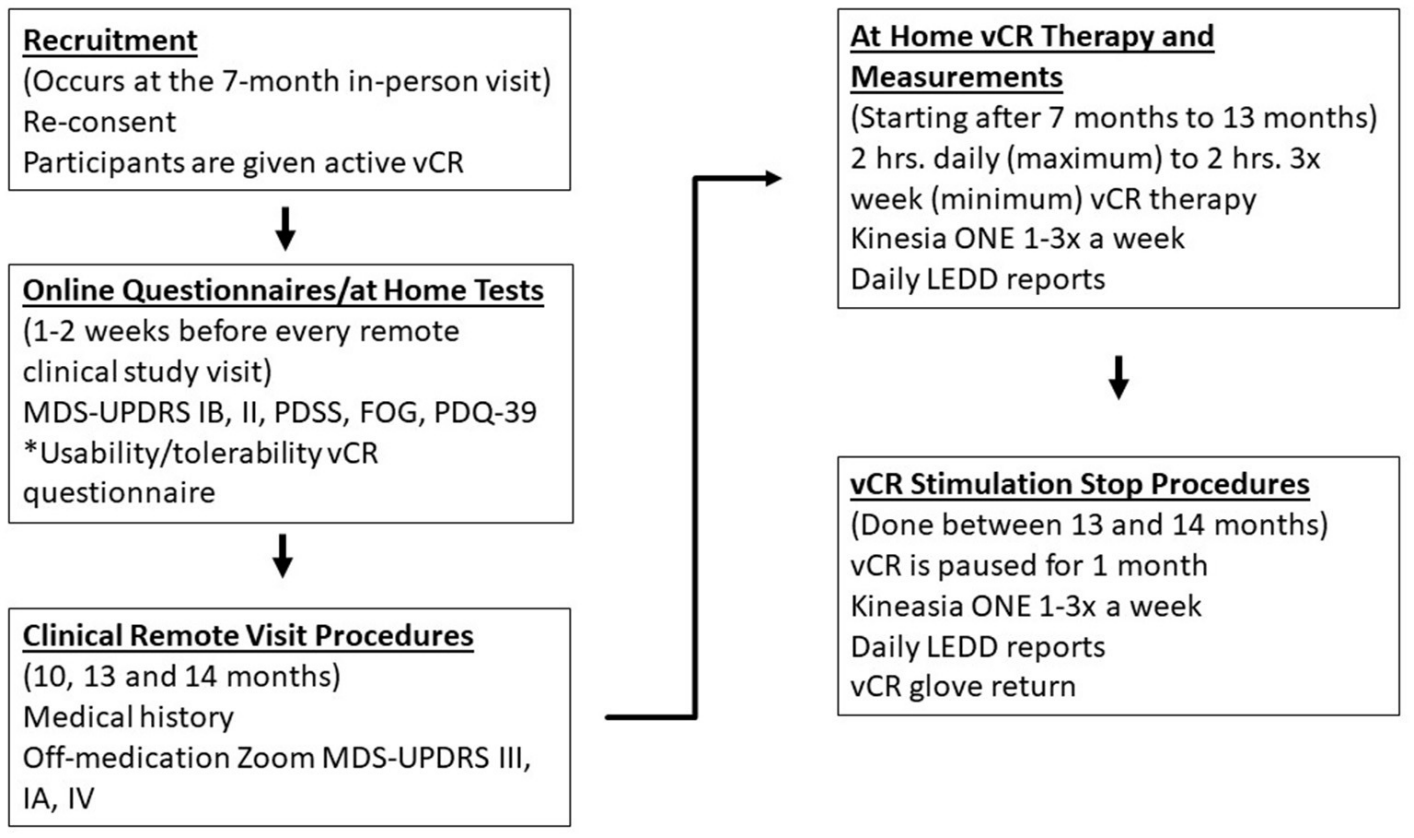
Depicts study 2 remote procedures that detail vCR dosing techniques and clinical data obtained. At the 7-month in-person visit, patients will be reconsented and will be sent home with active vCR. Patients will be instructed to stimulate between 2 h a day (maximum) to 2 h three times a week (minimum) from months 7 to 13, report their LEDD amount and perform the Kinesia ONE motor assessment one to three times a week to monitor motor ability throughout the entire trial. One to two weeks before every remote study visit, patients take the following questionnaires: MDS-UPDRS IB, II, PDSS, FOG, PDQ-39, and the Usability/Tolerability vCR device questionnaire. Remote study visits occur at 10, 13 months, and after a 1 month (14th month) pause in stimulation to assess long-lasting effects. During these remote study visits, patients are off medication and the study's treating neurologist gathers medical history and the study's main movement disorders evaluator assesses the remote MDS-UPDRS III, IA, and IV. *The Usability/Tolerability questionnaire is performed 1 week after the start of therapy and then 1–2 weeks before every study visit.

If the patient chooses to remain in the study, the following procedures will take place remotely in the patient's home. After the 7-month follow-up in study 1, the patient will be reconsented sent home with the vibrotactile device equipped with active vCR. The patient will stimulate as needed and as instructed using the parameters described above. Patients will take the online the PDQ-39, MDS-UPDRS parts IB and II, PDSS, FOG, and the usability and tolerability questionnaire of the vCR device. Patients previously in the sham condition will be given the usability and tolerability questionnaire 1 week after they start active vCR. Patients will complete all online questionnaires 1–2 weeks before every remote study visit. The patient will also continue to use the Kinesia ONE device one to three times a week so staff can monitor motor ability and report LEDD. The treating movement disorders neurologist will call patients after 1 month of stimulation to ask questions regarding the patients' treatment. At-home follow-up motor evaluations will take place at 10, 13, and 14 months *via* video meeting with the study's main MDS-UPDRS movement disorders evaluator. The study's treating movement disorders neurologist will additionally accompany the video call to check in on the patient. Depending on the type of medication, patients will go off medication (12–48 h) for these evaluations and perform the Kinesia ONE motor evaluation and the remote administration of the MDS-UPDRS IA, III, and IV (38). The remote MDS-UPDRS III will be our primary outcome measure, while all other measures are considered secondary. After 6 months of establishing a dosing regime, the patient will undergo a 1-month pause in stimulation and take his or her final online questionnaires and remote motor evaluation. The patient will mail his or her vCR device back to Stanford and will be thanked for his or her participation. For a detailed schematic of Study 2 events see Figure 4.

#### Statistical Analysis Study 2

The 15 patients receiving real vCR for 4 h a day in study 1 will be compared to the 15 patients who switch to real vCR in study 2 (previous sham patients). A 2 (4 h of daily vCR stimulation vs. decreased vCR stimulation) by 4 (baseline, 3, 6, and 7 months) mixed factorial ANOVA will be done individually on the MDS-UPDRS I, II, III, IV, with ratings for part III comprising of only remote tasks, LEDD, PDQ-39, FOG, PDSS, and Kinesia ONE ratings.

We hope to see no significant differences in evaluations taken from individuals receiving 4 h of stimulation and individuals receiving reduced vCR stimulation. This may suggest that between 2 h a day to 2 h three times a week of therapy is sufficient to drastically improve PD symptoms.

### Medication Withdrawal Procedures

In both studies, depending on the type of medication and, hence, its half-life, patients will withdraw from their medication for a maximum of 48 h prior to their off-medication assessments. Specifically, extended-release drugs: Mirapex extended release, Requip extended release and Neupro patches will be stopped 48 h prior to evaluations. Regular Mirapex, regular Requip, Sinement sustained or extended release and Rytary are stopped 24 h prior to off evaluations. Sinement immediate release, Comtan, Stalevo, Amantadine, Azilect, Selegiline, and Artane are stopped 12 h prior to off-medication evaluations.

### Adverse/Serious Events Reporting From the Vibrotactile Device

There are no formal statistics available on the vibrotactile stimulator's safety. Previous pre-clinical study patients have reported the glove as being tolerable with little to no side effects (17). There may be physical discomfort (e.g., pinching, numbing, skin indentations, etc.) associated with wearing the vibrotactile glove and study personnel will be vigilant for this unlikely possibility. In a previous study (17), patients reported a decrease in their medication over the course of vCR treatment. With this in mind, we hypothesize that PD patients with medication-induced dyskinesias might experience an increase of the medication-induced dyskinesias as vCR stimulation reduces the required medication dose, and as a result, patients may want to decrease their medication. If patients experience dyskinesia from the vibrotactile device, they are instructed to consult their neurologist about potential medication decreases and to not decrease their medication without consulting their doctor first. If patients and their neurologists decide to decrease their medication, patients are instructed to contact the study's treating movement disorders neurologist no later than 10 business days since medication changes. If in the event the patient experiences clinical worsening from the vibrotactile device, the patient is instructed to contact the study's treating movement disorders neurologist no later than 10 business days from the time of the event.

### Standard Operating Procedures and Training

For all evaluations, standard operating procedures (SOPs) have been developed to ensure uniformity between all study personnel. All study personnel received training of all protocol practices and use of equipment for their protocol roles.

## Discussion

This paper describes the study procedures for the study *Vibrotactile coordinated reset: a non-invasive treatment for Parkinson's disease*. This study is comprised of two types of study protocols which include Study 1, main phase double-blind sham-controlled study, and Study 2, second phase, remote dosing regimen within the same study participants who all receive active vCR. In both studies, vCR therapeutic benefits are examined by implementing motor and non-motor evaluations.

### Study 1

Testing vCR against a dedicated sham group will further assist in the true understanding of vCR's therapeutic motor benefits. A larger sample size may additionally aid in how vCR affects sub-motor types of PD, for example, tremor-dominant (TD), postural instability and gait difficulty dominant (PIGD-GD) and akinetic rigid types.

Non-motor related questionnaires and examinations such as sleep and smell have not yet been systematically studied in vCR experiments. Olfactory loss is commonly reported in PD patients, with some studies reporting ≥ 90% of patients with smell deficits (39, 40). The olfactory system is distinct, in that it has the unique capability to be activated by sniffing which by definition is a sensorimotor ability (41). Therefore, a therapy modulating sensorimotor areas of the brain (17) may have a positive impact on olfactory ability. Sleep disturbances are frequently reported in PD patients (42). Causes of sleep disturbances have been associated with nocturnal motor symptoms and dopaminergic medication (43). Given that vCR has positive benefits on the motor system and allows for a reduction of dopaminergic medications (17), we expect sleep to improve with vCR treatment. Speech and voice abnormalities are another area of interest simply because dopaminergic medication has been known to cause dysfluent speech (44–46) and traditional DBS can cause worsening of voice and speech (11). While the cause of voice and speech abnormalities is poorly understood, it is believed that improper integration of sensory and motor inputs due to dopamine loss within the striatum and basal ganglia can result in motor deficits that negatively affect subsystems related to speech motor control (47). Numerous studies have documented PD speech abnormalities related to sensorimotor deficits including errors in kinesthetic measurements (48), problems involving orofacial perception (49), and difficulties incorporating proprioceptive information during movement (50). Therefore, a therapy that targets the sensory and motor system and its interactions may have a positive benefit on speech and voice abnormalities in PD.

Using possible techniques such as the VTDT may aid in the understanding of vibratory sensory differences or abnormalities on a per patient basis which could help modify vCR patterns and parameters. Specifically, time as a dependent measure signifying the patient's vibratory temporal discrimination threshold may correlate to vCR effects. These measurements can then be used to modify vCR parameters on an individual basis so that patients receive the maximal benefit from vCR therapy. This could ultimately lead to a calibration-based personalized vCR therapy. Our VTDT is motivated by studies exploring somatosensory temporal discrimination in PD patients (51, 52). For instance, Conte et al. (51) used paired electrical stimuli delivered through surface skin electrodes. They found that somatosensory temporal discrimination threshold values were significantly greater in PD patients compared to in healthy subjects. In PD patients, dopamine reduced (i.e., partially restored) somatosensory temporal discrimination thresholds, whereas DBS delivered to the STN further degraded (i.e., increased) somatosensory temporal discrimination thresholds (51). In our pilot studies, we observed that in the course of the vCR therapy PD patients needed less dopaminergic medication (17). Accordingly, we hypothesize that vCR therapy may cause a cumulative and long-lasting reduction of somatosensory temporal discrimination thresholds assessed off medication.

In our VTDT, we deliver two vibratory bursts to the index and the middle finger. This is because the goal of vCR therapy is to reduce abnormal synaptic connectivity. We hypothesize that due to vCR treatment, unwanted synaptic connectivity and, hence, abnormally strong interactions between index and middle finger decrease so that sensory input from index and middle finger can be processed in a separated and, thus, more efficient manner. Effective CR stimulation requires that the overlap of stimulated neuronal sub-populations should not attain higher levels (17, 23, 53). Hence, there might be an intricate relationship between the vibration amplitude used for vCR and treatment outcome. For instance, the stronger the abnormal synaptic connectivity between neighboring fingers, the smaller the vibration amplitude should be. However, particularly weak vCR may be less effective since the desired vibration phase-locked neuronal activity may occur in only smaller portions of the sensory thalamus and the sensorimotor cortex [see (16, 17)]. Hence, vCR stimulation might be more favorable if delivered at vibration amplitudes adapted to the VTDT results. Accordingly, during the course of vCR treatment the optimal vibration amplitude might need to adapt using VTDT results. However, this remains to be shown, e.g., in a first step by correlating VTDT and therapeutic outcome.

Patients with PD suffer from impairments in sensorimotor integration (48, 54–56). EEG recordings specifically investigating sensorimotor activity are important in understanding how the sensory system interacts with the motor system in PD. Vibration alone activates cortical motor areas of the brain (27), with desynchronization of the alpha and beta band corresponding to increased sensorimotor activity (27, 28). Faster (button press) times to a visual cue have been associated with desynchronization of sensorimotor rhythm (SMR) within the beta band, while increases of SMR are associated with longer reaction times (57). In our previous study, we found that after 3 months of vCR treatment, PD patients displayed a decrease in high beta band power over the sensorimotor cortex while patients were at rest (17). Based on this finding, we expect to find similar decreases in beta band power in response to a vibratory cue. In addition, reduction in reaction times accompanied by reduced beta power activity over the sensorimotor cortex during the course of vCR treatment could serve as a possible indicator of increased sensorimotor integration.

The readiness potential (RP) is an event related potential (ERP) slow wave that begins 1–2 s preceding voluntary movement (58). The RP contains early and late components, with the early component reflecting preparation of movement and the later component related to motor execution (58). The early RP is thought to be generated from the supplementary motor area (SMA) while the late readiness potential is associated with activation of the primary motor cortex (59). Dysfunction of the SMA (60, 61) and lower amplitudes of the RP have been found in PD patients (62–65). For this study, increases in RP amplitude prior to button press during the course of vCR treatment could serve as an indicator of improvements in motor ability and SMA function. In addition, Contingent Negative Variation (CNV) is a slow wave cortical potential that is related to attention, expectancy, and motor preparation (66, 67). It occurs when a participant is presented with a cued stimulus (i.e., vibration or sound) that requires a motor response (59, 67). Patients with PD exhibit reduced CNV amplitude (65, 68). Increases in CNV amplitude could serve as a measure of improved preparation and execution of a motor response in PD patients treated with vCR.

### Study 2

Optimal therapeutic vCR outcome will require sufficient compliance. Accordingly, identifying a proper dosing regime is essential in providing patients with a reasonable number and duration of therapy sessions per week, such that these session times do not significantly interfere with their daily life. Motor and non-motor data of patients who received 4 h of daily active vCR stimulation for 6 months in study 1 will be compared to data from patients who received a lesser amount of vCR stimulation in study 2, which will further allow us to determine how much vCR stimulation is needed per week to produce maximal benefits. Our hope is that between 2 h of daily stimulation to 2 h daily three times a week will be sufficient to provide significant benefits that are equal to positive outcomes obtained from 4 h of stimulation a day. In our previous case study (17) we reduced one patient's daily 4-h dose of vCR therapy to 2 h three times a week. This patient had previously received 4 h of daily stimulation for 6 months. When the patient received a lower dose of vCR for 3 months, no substantial differences were found the patient's motor ability or medication intake. Specifically, the patient further improved as witnessed by his or her off-medication MDS-UPDRS part III score. Computationally, it was shown that long-term effects of CR stimulation do not only depend on stimulation duration. Rather, optimal dosing regimens with sufficient pausing in between CR epochs may cause long-lasting desynchronization even if CR stimulation is administered at particularly weak intensities rendering permanently delivered CR stimulation ineffective (69).

### Summary and Outlook

Our study protocol comprises two study phases: *Study 1* is a double blinded randomized sham-controlled proof-of-concept study which corresponds to a phase IIA trial of the pharmaceutical trial categorization (70). The goal of this study is to demonstrate clinical efficacy of vCR compared to sham stimulation. To ensure therapeutic dosage, in study 1 we will apply two times 2 h of vCR or sham stimulation per day, respectively. However, in one patient of our case studies, in total 2 h vCR per day were sufficient to cause pronounced therapeutic effects (17), indicating that a daily dose of 4 h may not be necessary.

*Study 2* aims to obtain knowledge of the therapeutic benefits of vCR stimulation at a reduced dosage regimen. To this end, sham patients from study 1 will be crossed over into active vCR for study 2, totaling 30 active patients for the dose finding study 2. All patients in study 2 will receive vCR at a dose ranging from 2 h a day (maximum daily dose) to 2 h a day three times a week (minimum weekly dose) for 6 months. Patients will select their actual weekly dose within this reduced dosage range depending on their individual needs, supposedly requiring less compliance and causing less interference with patients' day-to-day activities. Study 2 serves three purposes: (i) In the patients who received vCR in study 1, study 2 enables to collect data regarding safety, tolerability, and efficacy on a longer time scale by delivering vCR for in total 12 months instead of 6. (ii) Comparing the effects obtained in vCR patients in study 1 with the effects observed in the sham patients from study 1 crossing over to low-dose vCR in study 2 provides a dose finding comparison between low-dose and high-dose 6-month vCR therapy, similar to a phase IIB trial in terms of the pharmaceutical trial categories (70). (iii) In addition, in both patient groups (i.e., vCR vs. sham patients from study 1) therapeutic effects obtained in study 2 will be separately correlated with the integral amount of self-administered dose.

Depending on the results of studies 1 and 2, additional dose finding studies might be envisioned to further optimize and potentially reduce the dosing pattern as well as to further optimize the stimulation pattern, e.g., by increasing the temporal jitter of stimulus onsets used for noisy vCR (17).

## Conclusion

The aim of this study is to understand how vCR treatment affects a wide range of clinical symptoms associated with PD. We hypothesize that results obtained from this study will demonstrate clinical efficacy of our vCR therapy, procedure, and our investigational vibrotactile device for the purpose of acquiring FDA clearance.

## Ethics Statement

The study *Vibrotactile Coordinated Reset: a non-invasive treatment for Parkinson's Disease* was reviewed and approved by Stanford's Institutional Review Board. Written consent will be obtained from all patients prior to the start of study 1 and 2. All research staff has taken a Good Clinical Practice Course online.

## Author Contributions

BM and PT: designed vibrotactile glove system. KP and PT: wrote manuscript. All authors contributed to the overall design of the study protocol and revised/approved manuscript.

## Funding

This study was funded by Synergic Medical Technologies, Inc., (Agreement #: 59621).

## Conflict of Interest

RD has served as a clinical trials investigator for Impax Pharmaceuticals, Pharma2B, CALA Health, Axovant and Neurocrine Biosciences. PT works as consultant for Boston Scientific Neuromodulation and Gretap AG and is inventor on a number of patents for non-invasive neuromodulation. BM is employed by Engineering Acoustics who manufacture vibrotactile systems. This study received funding from Synergic Medical Technologies, Inc., The Parkinson Alliance and John A. Blume Foundation. Synergic Medical Technologies, Inc. was not involved in the collection, analysis, interpretation of data, the writing of this article or the decision to submit it for publication. We discussed the study design with Synergic Medical Technologies, Inc. and adjusted some aspects of the study design for compliance with the FDA approval process. The Parkinson Alliance and John A. Blume Foundation were not involved in the study design, collection, analysis, interpretation of data, the writing of this article or the decision to submit it for publication. No further disclosures. The remaining authors declare that the research was conducted in the absence of any commercial or financial relationships that could be construed as a potential conflict of interest.

## Publisher's Note

All claims expressed in this article are solely those of the authors and do not necessarily represent those of their affiliated organizations, or those of the publisher, the editors and the reviewers. Any product that may be evaluated in this article, or claim that may be made by its manufacturer, is not guaranteed or endorsed by the publisher.
